# Analysis of multiple‐herbicide resistant *Amaranthus palmeri* populations from Spain points to an introduction of the eccDNA from America

**DOI:** 10.1002/ps.70034

**Published:** 2025-07-10

**Authors:** Alfredo Manicardi, Germán Mora, André Lucas Simões Araujo, Todd A. Gaines, Jorge Lozano‐Juste, Joel Torra

**Affiliations:** ^1^ Department of Forest and Agricultural Sciences and Engineering Agrotecnio‐CERCA Center, University of Lleida Lleida Spain; ^2^ Instituto de Biología Molecular y Celular de Plantas, Universitat Politècnica de València, Consejo Superior de Investigaciones Científicas, IBMCP‐UPV‐CSIC Valencia Spain; ^3^ Department of Agricultural Biology Colorado State University Fort Collins Colorado USA

**Keywords:** palmer amaranth, ALS‐resistance, glyphosate‐resistance, extrachromosomal circular DNA, herbicide resistance

## Abstract

**BACKGROUND:**

The herbicide‐resistant invasive weed species *Amaranthus palmeri* threatens agricultural production and native plant ecology in Spain, as well as in other European countries. Understanding whether herbicide resistance alleles evolve *in situ* or are introduced via gene flow remains unclear. To address this, we characterized multiple resistance to acetolactate synthase (ALS)‐‐ and 5‐enolpyruvylshikimate‐3phosphate synthase (EPSPS)‐inhibiting herbicides in two Spanish *A. palmeri* populations at the plant level. Additionally, we analyzed the extra‐chromosomal circular DNA (eccDNA) to determine whether glyphosate resistance resulted from local selection pressure or was introduced by gene flow.

**RESULTS:**

Both populations exhibit individuals that survived both herbicide MoA, with multiple resistance mechanisms to ALS‐ and EPSPS‐inhibiting herbicides. Eight different *ALS* allele mutations were identified in resistant plants, including Pro‐197‐Ile, reported only in one species previously. Glyphosate resistance in the two populations is to the result of gene duplication mediated by eccDNA. Spanish and North American eccDNAs showed complete identity, with no single nucleotide polymorphisms (SNPs) found between the partial analyzed sequences of noncoding regions.

**CONCLUSION:**

We confirm for the first time in Europe resistance to ALS and EPSPS inhibitors at both the population and individual levels in two Spanish *A. palmeri* populations. The absence of SNPs in the eccDNA from Spanish populations compared to the reference American sequence and the presence of target‐site mutations in the *ALS* gene occurred without selective pressure from ALS herbicides, suggests that the origin of resistance traits may have evolved elsewhere and been introduced from the place of origin to Spain. However, it is important to note that the limited number of populations studied and the partial sequencing of eccDNA do not provide definitive confirmation of the exact origins of resistance mechanisms. This work raises concerns about the arrival of this and potentially other new herbicide‐resistant *A. palmeri* populations in Europe posing challenges for management. © 2025 The Author(s). *Pest Management Science* published by John Wiley & Sons Ltd on behalf of Society of Chemical Industry.

## Graphical abstract

In this study, multiple herbicide resistant mechanisms have been confirmed in two *Amaranthus palmeri* populations from Spain that survived glyphosate‐ and ALS‐inhibitor herbicides. Our data suggest that the eccDNA and the mutations identified in the ALS gene are the result of an evolutionary event that occurred outside Spain.

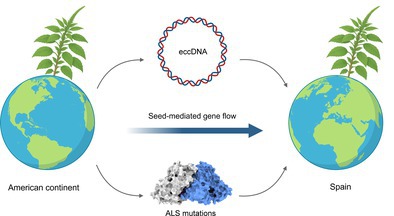

## 1 INTRODUCTION

The field of weed science has revolutionized agricultural practices, especially since the introduction of selective herbicides in the mid‐20th Century. Chemical weed management enhanced agricultural productivity and minimized labor‐intensive weed removal processes.^1^, ^2^ However, the continuous reliance on herbicides with the same mode of action (MoA) as the singular weed management strategy has led to the evolution of herbicide‐resistant (HR) weed populations.^3^, ^4^ Weeds can invade new habitats as a consequence of human activity, such as movement of contaminated seeds or soil, or by natural dispersal, such as bird migrations, and introduce their genetic traits into the new environment.^5^, ^6^, ^7^ Significant risks to local agricultural systems occur if introduced individuals of an invasive species carry herbicide resistance traits.

*Amaranthus palmeri* S. Watson is a perfect example of a highly problematic invasive weed species. Thanks to a rapid growth rate, high adaptability to different environments and prolific seed production, this species is highly competitive with crops, and it can drastically reduce yield production.^8^, ^9^ In the United States, this species ranks among the most difficult weeds to manage, primarily owing to its remarkable capacity for developing herbicide resistance. As a dioecious plant, it relies on obligate outcrossing for reproduction, which facilitates the rapid dissemination of herbicide‐resistance genes across populations and countries.^9^ To date, resistance to nine different herbicide MoAs has been reported for *A. palmeri*, including 5‐enolpyruvylshikimate‐3phosphate synthase (EPSPS),^10^ acetolactate synthase (ALS),^11^ photosystem II,^12^ 4hydroxyphenylpyruvate dioxygenase,^13^ protoporphyrinogen oxidase,^13^, ^14^ very long chain fatty acid elongase,^15^ microtubule‐inhibitor herbicides,^16^ synthetic auxins^17^ and glutamine synthetase.^18^, ^19^ Chemical control of *A. palmeri* is particularly difficult because this species can accumulate multiple resistance mechanisms to different herbicide MoAs within a single individual.^20^, ^21^ Multiple herbicide resistance may result from co‐evolution of both nontarget‐site and/or target‐site resistant mechanisms at plant level. Previous reports have described populations able to gather resistance mechanisms for up to six different MoAs including ALS‐, PS II‐, HPPD‐, PPO‐ and EPSPS‐inhibiting herbicides.^22^

*Amaranthus palmeri* has quickly spread outside is native region (southwestern US and north of Mexico) threatening local food production into North and South America, South Africa, Japan and the Mediterranean region.^9^, ^23^, ^24^, ^25^, ^26^, ^27^ Previous reports have tried to identify the cause of introduction of this species into new environments, pointing out import–export trades of grains and seed movement by machinery as the main reasons for long‐ and short‐range spread, respectively.^28^, ^29^ Seeds originating from environments with strong herbicide selection pressure may introduce genes for herbicide resistance, thereby speeding up the local development of resistant populations.^30^ The extensive use of genetically engineered crops resistant to ALS and EPSPS inhibitors throughout North and South America has considerably increased the selection pressure from these MoAs accelerating the evolution of herbicide‐resistant *A. palmeri* biotypes.^31^ These MoAs account for the highest number of herbicide resistance cases for this species worldwide.^3^, ^10^, ^28^, ^29^

ALS‐inhibiting herbicides bind to the ALS enzyme inhibiting branched chain amino acid biosynthesis. This inhibition disrupts protein synthesis and essential metabolic processes, blocking plant growth and eventually leading to plant death. Multiple *A. palmeri* populations have been reported worldwide to be resistant to the ALS inhibitors owing to different point mutations in the *ALS* gene.^11^, ^20^, ^21^, ^22^, ^23^, ^24^ Resistant populations to ALS‐inhibitors have recently been reported in several European and Mediterranean countries,^25^, ^26^, ^27^, ^28^ with Spain as one of the most affected territories.^29^ Glyphosate kills plants by inhibiting EPSPS, a crucial enzyme in the biosynthesis of aromatic amino acids. *A. palmeri* has evolved resistance to glyphosate by increasing *EPSPS* gene copy number.^10^, ^31^ Target‐site mutations in *EPSPS* are rare because they affect the enzyme‐substrate complex with 2‐phosphoenolpyruvate (PEP), which is crucial for EPSPS biochemical efficiency.^32^, ^33^ Increased *EPSPS* copy number in *A. palmeri* is controlled by a ~400‐kb extrachromosomal circular DNA (eccDNA).^34^, ^35^, ^36^ This replicon consists of the *EPSPS* gene and other coding genes related to abiotic stress resistance (e.g. *NAC* and *HSC70*) and a reverse transcriptase along with an autonomous replication sequence.^37^ Genetic studies on resistant *A. palmeri* populations from North and South America suggest a unique shared origin based on the high similarity of the *EPSPS* eccDNA sequence found in different populations from USA, Brazil and Uruguay.^23^, ^37^, ^38^, ^39^ In a recent study on Spanish and Italian populations, we demonstrated that ALS‐inhibitor resistance evolved in separate evolutionary events outside these countries with different origins of introduction.^30^ This increases the risk of importing resistance to other MoAs, such as glyphosate, further complicating the management of this species. We also described a Spanish population resistant to glyphosate with no point mutations in the *EPSPS* gene, and the presence of the *EPSPS* eccDNA that could explain resistance to glyphosate.^40^ We are uncertain whether glyphosate resistance evolved *de novo* in Spain or elsewhere. Finally, because new seeds are constantly imported and considering their ability to evolve resistance to multiple MoAs, it remains uncertain whether Spanish *A. palmeri* biotypes already have evolved multiple herbicide resistance to glyphosate and ALS inhibitors. This work aims to study two Spanish populations of *A. palmeri* from Catalonia (Spain) with the purpose to (i) investigate multiple resistance to EPSPS‐ and ALS‐inhibiting herbicides at the population and individual level, (ii) characterize the mechanisms conferring resistance to both MoAs, and (iii) to unravel the *in situ*/*ex situ* generation of the eccDNA comparing the similarity between Spanish eccDNA and the American reference sequence.

## 2 MATERIALS AND METHODS

### 2.1 Plant material

Three *A. palmeri* populations were included in this study. Two were collected in roadsides in Montblanc (MB) (41° 22′ N, 1° 9′  E) and in Tarragona (TA) (41.1189° N, 1.2445° E) in NE Spain, and one from North Carolina, USA, which was used as a sensitive control, the wild‐type (WT).^41^ To date there is no standard sensitive population from Spain, and for this reason an American one was used. Seeds were collected from ≥10 different mother plants, cleaned and stored at room temperature.

### 2.2 ALS‐inhibitor treatment

Bioassays were performed in summer season in a tunnel located in the University of Lleida (41° 37′ N, 0° 35′ E). Temperatures ranged from 24 °C to 26 °C at night and from 26 °C to 35 °C during the day. Seeds were first sown in aluminum boxes (30 × 10 × 5 cm) with peat, watered and placed in a germination chamber with a 16‐h:8 h, light:dark cycle at 29 °C and an 8‐h:16‐h, light:dark cycle at 22 °C. After 5 days, 20 seedlings per population were transplanted into plastic trays (325 × 265 × 95 cm^3^) with 60% silty loam soil, 15% perlite, 10% peat mix and 15% peat on top, and watered daily. At the 4–5 leaf stage, plants were treated with a single dose of thifensulfuron‐methyl (THIF) [Harmony 50 SX (DuPont™, Wilmington, DW, USA) 500 g ai kg^−1^] and imazamox (IMA) [Tuareg® (DuPont™) 40 g ai L^−1^ + 50% w/v Dash, wetting agent] at recommended doses of 6 and 40 g ai ha^−1^, respectively. Herbicides were applied using a precision bench sprayer equipped with three flat‐fan hydraulic nozzles (11 002; Teejet®, Glendale Heights, IL, USA), delivering 300 L ha^−1^ at a pressure of 215 kPa and ≈0.75 m s^−1^ speed. The design was completely randomized, and each treatment had three replicates, for which 20 plants per replicates were used (*n* = 60 plants per dose). Untreated replicates were used as control for each population. Four weeks after herbicide application, survival rate was determined.

### 2.3 *Glyphosate* dose–response treatment

Plants were grown as described in the previous section. During the 4–5 leaf stage plants were exposed to glyphosate treatment [Roundup® SC (Bayer CropScience, Leverkusen, Germany) 360 g ai L^−1^) with the following rates: 0, 25, 50, 100, 200, 400, 800 g ae ha^−1^ for WT, and 100, 200, 400, 800, 1600, 3200 g ae ha^−1^ for MB and TA. We selected these rates because WT was completely controlled at 400 g ae ha^−1^, as validated previously.^40^ The experiment followed a completely randomized design. For each population, the six different herbicide doses were tested in triplicate, using 20 plants per treatment (*n* = 360). Four weeks after herbicide application, survival rate and fresh weight (FW) were determined. The FW and survival data were fitted to a nonlinear log‐logistic regression model using sigma plot 12.0 (SPSS Inc., Chicago, IL, USA) statistical software, which allowed the estimation of herbicide dosages required to reduce plant growth by 50% (GR_50_) and to cause 50% mortality in a population (LD_50_) compared to the untreated control.^42^ Afterwards, the resistant factor (RF) was calculated, defined as the GR_50_:LD_50_ ratio of the resistant (R) weed population compared to that of a susceptible (S) population (ratio R to S).

### 2.4 Glyphosate single‐dose treatment

In order to compare Spanish and American *EPSPS cassette* (eccDNA) sequence we performed a single‐dose experiment to select plants from TA and MB populations containing the replicon. Seeds were collected from ≥10 different mother plants per population and grown as described before. Plants were treated at 4–5 leaf stage with 400 g ae ha^−1^ glyphosate. The design was completely randomized, and each treatment was performed in triplicate, *n* = 60 (number of plants per treatment). The decision to use plants from different mother plants was made to enhance genetic variation.

### 2.5 Sequential application of glyphosate and ALS inhibitors

In order to assess multiple resistance to EPSPS and ALS inhibitors at the plant level in glasshouse conditions, a sequential application of single doses of glyphosate and THIF was carried out. Plants were grown as described previously. They were treated with glyphosate at a rate of 400 g ae ha^−1^ at the 4–5 leaf stage, followed by a THIF treatment at 6 g ai ha^−1^ 21 days after the initial glyphosate application. The design was completely randomized, and each treatment was performed in triplicate, *n* = 60 (number of plants per treatment). The WT population was included in each treatment. Four weeks after herbicide application, survival rate was determined.

### 2.6 DNA extraction

Fresh leaves were collected from plants that survived all herbicides (IMA, THIF and GLY) treatment and stored at −80 °C until genomic DNA extraction was performed. Briefly, leaves (≈100 mg) were ground and kept cold with liquid nitrogen. Next, 375 μL 2× lysis buffer (0.6 m NaCl, 0.1 m Tris–HCl pH 8.0, 40 mm EDTA pH 8.0, 4% Sarcosyl and 1% SDS) and 2 m Urea were added, and the samples were briefly vortexed. Subsequently, 750 μL of 25:24:1 phenol‐chloroform‐isoamyl alcohol was added, mixed by inversion, and centrifuged for 15 min at 14 000 rpm at room temperature (RT). The aqueous phase was collected, and DNA was precipitated using 0.7× volume of cold (−20 °C) 2‐propanol. The mixture was centrifuged for 15 min at 14 000 rpm at 4 °C. After removing the supernatant, the resulting pellets were washed twice with 70% ethanol, centrifuged for 5 min at 14 000 rpm at 4 °C, and then dried for 15 min at RT. The dried pellets were reconstituted in 100 μL resuspension buffer (1 m Tris‐HCl + 30 μg mL^−1^ RNAsa A). DNA concentration and quality were assessed using a NanoDrop 2000 spectrophotometer (Thermo Fisher Scientific, Waltham, MA, USA) and DNA integrity by agarose gel electrophoresis.

### 2.7 PCR and sequencing of the *ALS* gene

DNA was extracted from a total of 20 and 24 individuals from MB and TA, respectively, that survived ALS‐inhibitor treatment [Table 2(a),(b)] and were used for this analysis. PCR were conducted in a 50 μL mixture, containing 5 μL of 5× buffer, 0.2 mm dNTPs mix, 10 μm each of forward and reverse primers, 0.5 μL Phusion DNA Polymerase (Thermo Fisher Scientific) and 100 ng DNA. Primers Ap_3F and Ap_4R were used to amplify the C‐, A‐ and D‐ domain (CAD) region^22^ under these cycling conditions: 95 °C for 5 min; 35 cycles of 95 °C for 30 s, 58 °C for 30 s, 72 °C for 54 s and a final 72 °C extension for 5 min (Table S3). Primers Ap_2F and Ap_2R amplified the BE region under similar cycling conditions but with a 72 °C extension for 21 s (Table S2). After amplification, the PCR products were purified using a PCR Clean‐up kit (FavorPrep) according to the manufacturer's instructions. Sanger sequencing was performed at the sequencing facility of IBMCP (Valencia, Spain), using both forward and revers primers, and sequences were visualized and aligned using geneious prime® 2023.2.1.

### 2.8 PCR and sequencing of the *EPSPS* gene

DNA was extracted from a total of 20 individuals from MB and TA, that each survived 400 g ae ha^−1^ glyphosate as described previously (*n* = 40). Gene amplification and PCR product purification were conducted as detailed before, except for the annealing temperature and amplification time, which were set at 61 °C and 15 s, respectively (Table S3). Using the primers EGF and EGR (Table S1) a 196‐bp DNA fragment from the conserved TAP region of the *EPSPS* gene^41^ was amplified by PCR and Sanger‐sequenced.

### 2.9 *EPSPS* copy number determination

A total of 20 individuals from MB and TA, respectively, that survived glyphosate treatments were used for this analysis. Twenty‐five days after dose–response application with glyphosate, tissues from surviving MB and TA plants together with three untreated WT plants were collected. DNA was extracted as mentioned earlier and quantitative real‐time polymerase chain reaction (qRT‐PCR) was performed to evaluate the relative *EPSPS* gene copy number and normalized to β‐tubulin,^35^ using primers: EPSPS_F, EPSPS_R3, βtubulin_F and β‐tubulin_R (Table S1). Each qRT‐PCR reaction contained 20 μL, comprising 10 μL SYBR Green Supermix (NZY Tech), 1 μL at 3.3 μm of forward and reverse primers, and 100 ng gDNA. Water was used as negative control. Data analysis was performed using the 2^−ΔΔCt^ method,^40^ expressing the genomic copy number of *EPSPS* relative to *β‐tubulin*, and the *EPSPS* copy number was relative to the susceptible samples. Each sample was assessed using 10 biological replicates for TA and MB populations, and three for WT and three technical replicates for all the populations.

### 2.10 *EPSPS* cassette partial amplification and sequencing

Twenty‐five days after treatment, PCR and Sanger sequencing analysis was conducted on three distinct noncoding regions (A, C and E) of the eccDNA from DNA samples extracted as detailed before from 20 individuals from both TA and MB populations (*n* = 40) survived a single glyphosate dose. The three regions of the *EPSPS* cassette (called A, C and E) were amplified and then sequenced using primers: eccA_F, eccA_R, eccC_F, eccC_R, eccE_F and eccE_R (Table S1) for the amplification of regions A, C and E, respectively [Fig. 4(b),(c)]. The regions are located as follows: region A between position 77 489 and 79 123; region C between 233 751 and 253 263; and region E between 288 232 and 289 218. All primers were designed by Molin and colleagues.^38^ Reactions were made in a 40 μL mix consisting of 1 μL of 100 ng DNA, 1.6 μL of 10 μm primers, 20 μL EconoTaq Plus Green 2× Master Mix (Biosearch technologies, Teddington, UK) and 16 μL H_2_O. Cycling conditions for eccA and eccC primer pairs were 94 °C for 2 min, 30 cycles of 94 °C for 30 s, 60 °C for 30 s, 72 °C for 2 min and 72 °C for 5 min. For eccE primers the annealing temperature was 52 °C and extension time was 1 min. After amplification, PCR products were sent for purification and Sanger sequencing to Genewiz (115 Corporate Boulevard, South Plainfield, USA). Sequences were visualized and aligned to an *EPSPS* cassette reference sequence (NCBI: MT025716.1) using geneious prime® 2023.2.1.

### 2.11 Multiple resistance at the plant level

In order to characterize the multiple resistance mechanisms to *ALS* and *EPSPS* inhibitors at the plant level, 10 individuals were selected from each population (*n* = 20) that survived a single glyphosate dose (400 g ae ha^−1^) and were confirmed to possess the *EPSPS cassette*. These selected individuals were used for partial amplification of the *ALS* gene as described earlier.

## 3 RESULTS

### 3.1 Resistance patterns to ALS and EPSPS inhibitors

**Figure 1:**
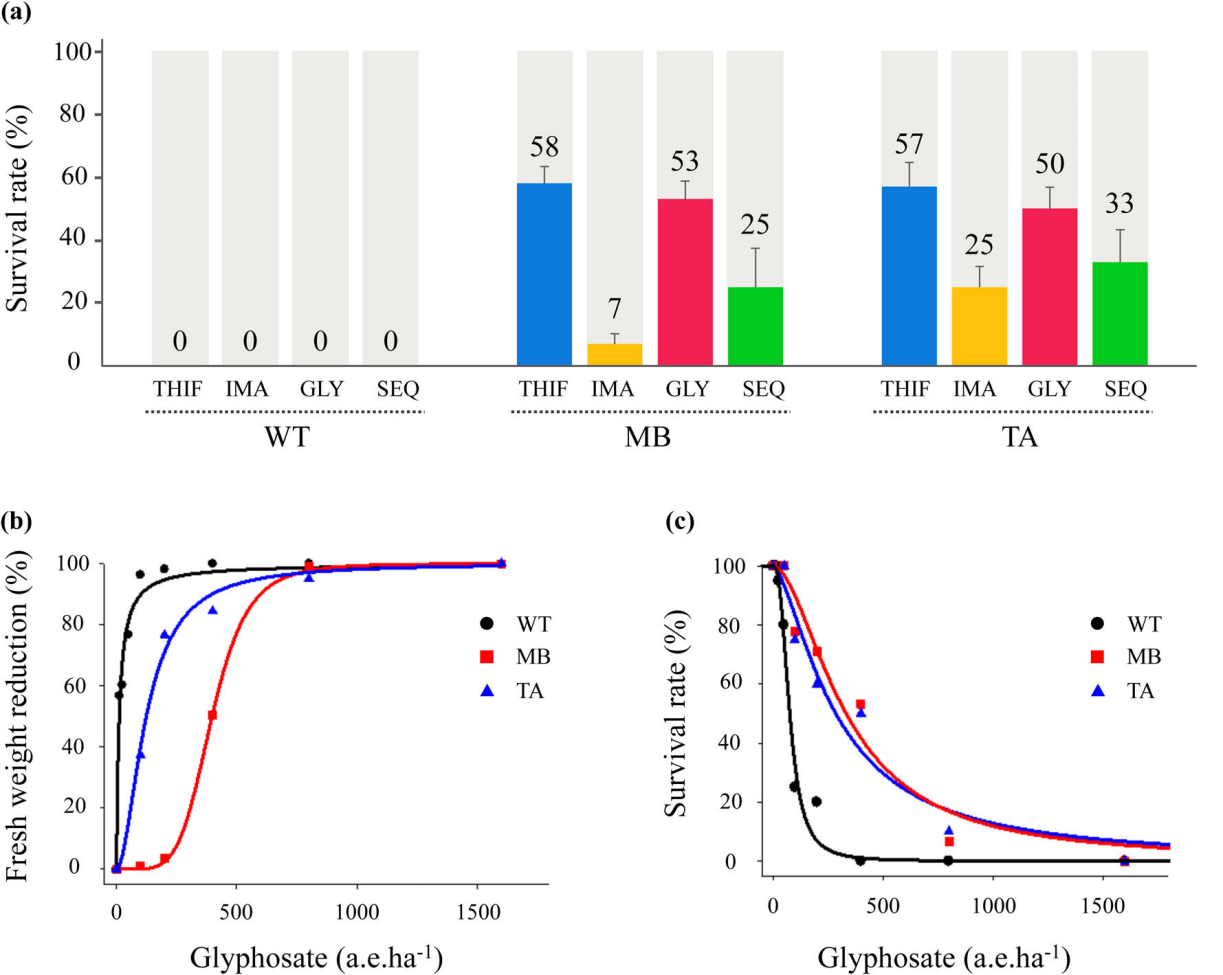
(a) Survival rates (%) of resistant *Amaranthus palmeri* populations TA and MB and the susceptible (WT) to ALS‐inhibitors thifensulfuron‐methyl (‘THIF’, blue bars) and imazamox (‘IMA’, yellow bars), glyphosate (400 g ae ha^−1^ ‘GLY’, red bars) and to the sequential application of glyphosate followed by thifensulfuron‐methyl (‘SEQ’, green bars). The values represent the mean of three replicates, and vertical bars the standard error. Regression curves of doseresponse to glyphosate (b) GR_50_ (expressed as % reduction in fresh weight) and (c) LD_50_ (expressed as % survival).

In order to study the resistance pattern of two Spanish *A. palmeri* populations, we first carried out applications of the ALS inhibitors THIF and IMA. The WT population was completely controlled by THIF and IMA applications, with 0% survival for both herbicides. The MB and TA populations had individuals that survived both treatments [Fig. 1(a)]. More individuals in each population were resistant to THIF than to IMA. The survival rate for THIF ranged from 57% to 58% for MB and TA populations, respectively, whereas the survival rate ranged from 7% to 25% for IMA [Fig. 1(a)]. After confirming resistance at the individual level to ALS‐inhibiting herbicides in the TA and MB populations, we extended our study to determine multiple resistance of both populations to glyphosate. Glyphosate applied at half‐recommended field dose (400 g ea ha^−1^) was sufficient to control 100% of the WT individuals, whereas 53% and 50% of the MB and TA populations, respectively, survived this glyphosate application [Fig. 1(a)]. Glyphosate dose–response curves for biomass reduction indicated similar resistance levels for both MB and TA populations. The GR_50_ (herbicide dose to reduce biomass by 50%) and LD_50_ (dose to cause 50% mortality) for MB and TA were 397.8 and 124.4 g ae ha^−1^, and 332.6 and 285.5 g ae ha^−1^, respectively, compared to the WT values of 12.0 g ea ha^−1^ (GR_50_) and 76.1 g ae ha^−1^ (LD_50_). RFs for FW reduction (GR_50_) were 33 and for survival rate (LD_50_) were 10.3 and 64.4, and 3.7 and 4.4, for MB and TA, respectively (Table 1). To understand if individuals of the MB and TA populations were resistant to both herbicide families (ALS and EPSPS inhibitors) simultaneously we performed the sequential application of glyphosate and ALS inhibitors. Herbicides in sequential application completely controlled the WT population 4 weeks after the first application with glyphosate (0% survival). By contrast, both MB and TA populations had multiple resistant individuals with survival values of 25% and 33%, respectively, for glyphosate followed by the ALS‐inhibitor THIF [Fig. 1(a)].

**Table 1:** Log‐logistic equation parameters of dose–response regression curves of survival and fresh weight in two populations of sensitive (WT) and resistant (MB and TA) populations treated with glyphosate. LD_50_ and GR_50_ represent the concentrations of herbicide required to decrease survival and fresh weight by 50%, respectively. Their relative standard error is reported in parentheses. Resistance factors (RF = R:S) is the ratio between resistant and susceptible GR_50_ or LD_50_. *P* is the level log significance of the nonlinear model

	Population	GR_50_ (g ae ha^−1^)	*P*‐value	Slope	RF
Fresh weight reduction	WT	12.0 (±4.9)	<0.0001	1.0	‐
	MB	397.8 (±1)	<0.0001	5.4	33.0
	TA	124.4 (±4.3)	<0.0001	1.9	10.3

	Population	LD_50_ (g ae ha^−1^)	*P*‐value	Slope	RF
Survival rate	WT	76.1 (± 5.9)	<0.0001	−2.7	‐
	MB	332.6 (± 9.2)	0.0001	−2	4.4
	TA	285.5 (± 8.6)	0.0001	−1.5	3.7

### 3.2 Resistance mechanism for ALS inhibitors

**Figure 2:**
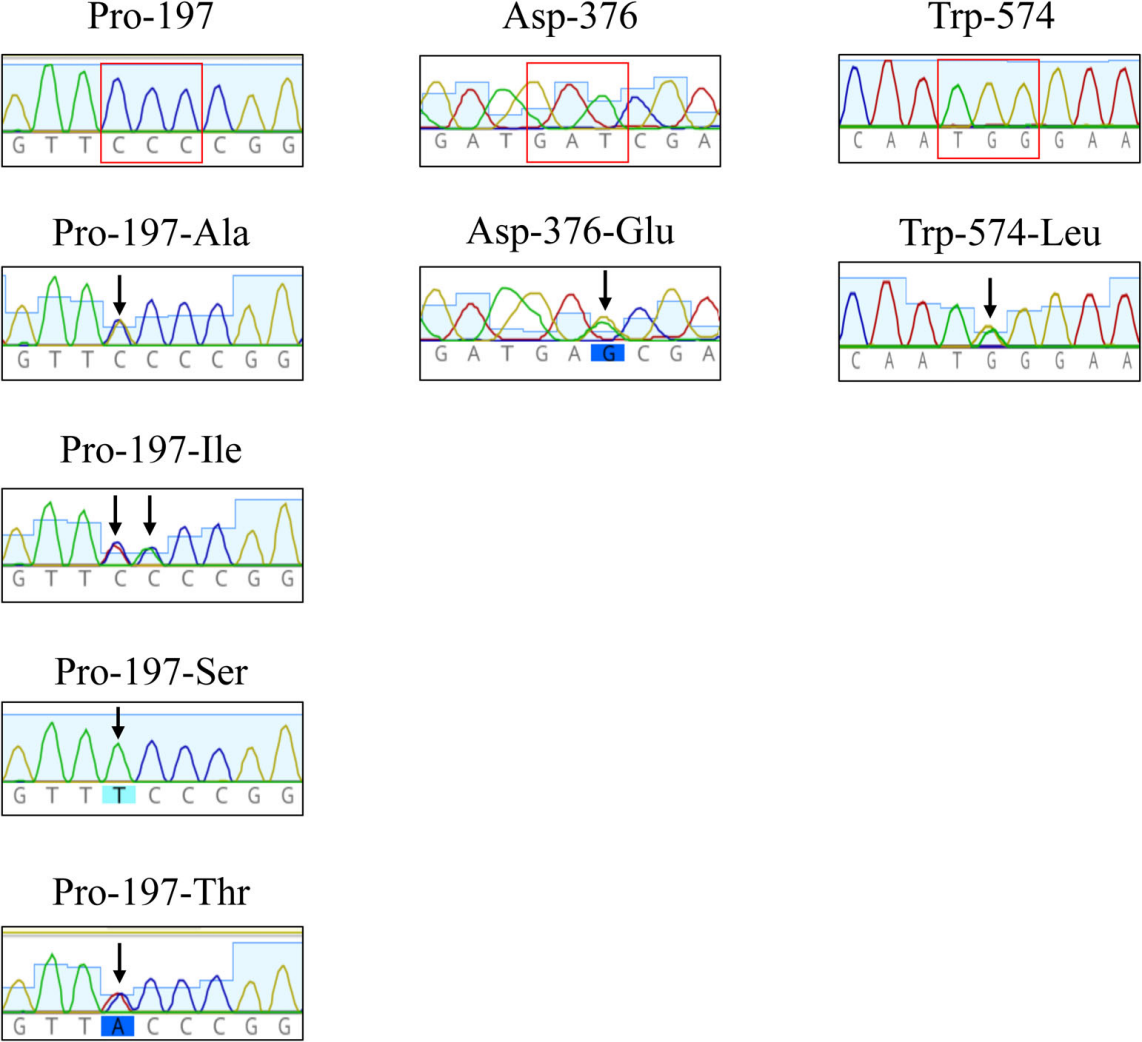
Chromatogram details for all of the different *ALS* mutations found in this work. The wild‐type version for each mutations at codons Pro‐197, Asp‐376 and Trp‐574 are highlighted in red (accession no. KY781923). The different amino acid changes are marked with a black arrow and detailed as followed: CCC encodes the amino acid proline (Pro‐197) at codon 197, whereas GCC encodes alanine (‐Ala), ATC an isoleucine (‐Ile), TCC encodes serine (‐Ser) and ACC encodes threonine (‐Thr); GAT codon encodes aspartic acid (Asp‐376) whereas GAA encodes glutamic acid (−Glu); TGG codon encodes the amino acid tryptophan (Trp‐574), whereas TTG encodes leucine (−Leu). The double peak indicates that the plant is heterozygous.

Resistance to ALS inhibitors in *A. palmeri* is often associated with amino acid changes at the herbicide binding site. To identify mutations in the ApALS gene, MB, TA and WT populations were analyzed by PCR followed by sequencing. Twenty and 23 individual plants surviving ALS inhibitors from the MB and TA populations, respectively, were analyzed. All sequences were aligned to the ApALS sensitive reference gene (accession no. MT583816.1). In both populations, we found different amino acid substitutions previously shown to confer resistance to ALS inhibitors.^43^ We identified amino acid changes at three ALS positions: Pro‐197, Asp‐376 and Trp‐574 (Table 2). The Pro‐197‐Ser mutation was identified in 13 plants surviving THIF (63%) from the MB population [Table 2(a)] and six (37.5%) from the TA population [Table 2(b)]. Five plants resistant to THIF from MB (2.6%) had the Pro‐197‐Thr mutation, of which four also exhibited the Asp‐376‐Glu mutation in the same analyzed sample. Only two plants had these mutations individually (one plant with only Pro‐197‐Thr and one with only Asp327 376‐Glu). One plant from the TA population survived IMA and had the Pro‐197‐Ile mutation. The Trp‐574‐Leu mutation was found alone in only one plant (0.5%) that survived IMA in MB and in eight in TA (50%). Generally, plants surviving IMA had only one known mutation in the analyzed *ALS* gene sequence. All plants with one of these mutations were heterozygous as shown in Fig. 2.

**Table 2:** Comprehensive list detailing all target‐site mutations identified in surviving plant from both the (a) MB and (b) TA populations to ALS inhibitors imazamox (IMA) and thifensulfuron methyl (THIF). At the end of each table, the total number of biotypes having a particular mutation is presented, expressed as a ratio of mutated biotypes over the total number of plants analyzed. ‘N.S.’ Sanger sequencing did not produce a clear sequence owing to technical problems

(a)					
MontBlanc (MB)					
Herbicide treatment	SampleID	Pro‐197‐Ser	Pro‐197‐Thr	Asp‐376‐Glu	Trp‐574‐Leu
IMA	MB_18	✓			
	MB_19			✓	
	MB_20				✓
THIF	MB_1	✓			
	MB_2	✓			
	MB_3		✓		
	MB_4	✓			
	MB_5		✓	✓	
	MB_6	✓			
	MB_7	✓			
	MB_8		✓	✓	
	MB_9	✓			
	MB_10	✓			
	MB_11	✓			
	MB_12	✓			
	MB_13		✓	✓	
	MB_14	✓			
	MB_15	✓			
	MB_16	✓			
	MB_17		✓	✓	
Total		13/20	5/20	5/20	1/20

(b)					
Tarragona (TA)					
Herbicide treatment	Sample ID	Pro‐197‐Ser	Pro‐197‐Thr	Pro‐197‐Ile	Trp‐574‐Leu
IMA	TA_1			✓	✓
	TA_2	✓			
	TA_3				✓
	TA_4				✓
	TA_5				✓
	TA_6				✓
	TA_7				✓
	TA_8				✓
	TA_9				✓
THIF	TA_12	✓			
	TA_13	✓			
	TA_14	✓			
	TA_15	✓			
	TA_16	✓			
	TA_17				
	TA_18				✓
	TA_19	N.S.			
	TA_20	N.S.			
	TA_21	N.S.			
	TA_22	N.S.			
	TA_23	N.S.			
	TA_24	N.S.			
	TA_25				✓
Total		6/23	0/23	1/23	10/23

In order to further characterize the plants found in our populations that were ALS‐inhibitor cross‐resistant (resistant to THIF and IMA) and also glyphosate‐resistant, we sequenced the *ALS* gene in 10 plants per population that survived a single glyphosate dose (Table 3). We found multiple amino acid substitutions at three different ApALS amino acid positions: Pro‐197, Asp‐376 and Trp‐574. A total of six plants (60%) from MB and seven (70%) from TA had a change at Pro‐197 as follows: six plants had 197‐Thr (four in MB, two in TA), four had 197‐Ser (two plants for each population), two had 197‐Ile (both from TA) and one had 197‐Ala (from TA). In the MB population, a Glu‐376‐Asp amino acid change was observed in four plants, whereas this mutation was not found in any TA individuals. Finally, one plant from MB and three from TA had a mutation for Trp‐574Leu. We also found plants combining two amino acid changes. Pro‐197‐Thr/Glu‐376Asp (MB) and Pro‐197‐Thr/Trp‐574‐Leu (TA).

**Table 3:** Comprehensive list detailing all target‐site mutations in the *ALS* gene identified in surviving plant from both the (a) MB and (b) TA populations to a single dose of glyphosate. At the end of each table, the total number of biotypes having a particular mutation is presented, expressed as the ratio of mutated biotypes to the total number of plants analyzed

(a)					
MontBlanc (MB)					
Herbicide treatment	Sample ID	Pro‐197‐Ser	Pro‐197‐Thr	Asp‐376‐Glu	Trp‐574‐Leu
GLY	MB_A1				
	MB_A2		✓		
	MB_A3			✓	
	MB_A4		✓	✓	
	MB_A5				✓
	MB_B1	✓			
	MB_B2		✓	✓	
	MB_B3		✓		
	MB_B4		✓	✓	
	MB_B5	✓			
Total		2/10	5/10	4/20	1/10

(b)						
Tarragona (TA)						
Herbicide treatment	Sample ID	Pro‐197‐Ser	Pro‐197‐Thr	Pro‐197‐Ile	Pro‐197‐Ala	Trp‐574‐Leu
GLY	TA_A1					✓
	TA_A3	✓				✓
	TA_A4					✓
	TA_A5					
	TA_A6			✓		
	TA_B1				✓	
	TA_B2	✓				
	TA_B3		✓			
	TA_B4			✓		
	TA_B5	✓				
Total		3/10	1/10	2/10	1/10	3/10

### 3.3 Resistance mechanism to EPSPS inhibitors

**Figure 3:**
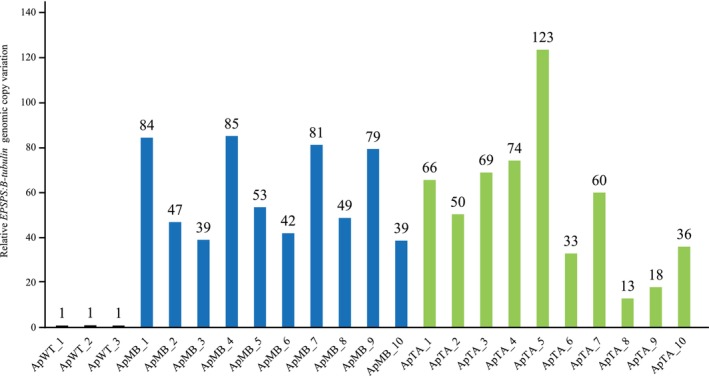
EPSPS genomic copy number in glyphosate‐susceptible (‘WT’, black bars) and glyphosate‐resistant populations MB (‘MB’, blue bars) and TA (‘TA’, green bars) *Amaranthus palmeri* populations. WT samples were collected from three untreated individuals. The EPSPS copy number is relative to the susceptible samples. MB and TA biotypes survived 400 g glyphosate ae ha^−1^. Data in qRT‐PCR were normalized using β‐tubulin as a reference gene.

Glyphosate surviving plants from both TA and MB populations were selected to characterize target‐site resistant (TSR) mechanisms. Mutations in the *EPSPS* gene region TAP‐ITV were studied using primers EGF and EGR (Table S1) for both untreated WT plants and those surviving from MB and TA populations treated with glyphosate. Sanger sequencing confirmed the absence of point mutations within the TAP‐ITV domain in any of the obtained sequences from MB, TA and WT populations. *EPSPS* copy number from glyphosate‐surviving plants was compared with three WT untreated plants by qRT‐PCR. Using *β‐tubulin* as a reference gene, we identified *EPSPS* gene copy numbers within the range of 13–123 in *A. palmeri* plants that exhibited resistance to glyphosate application, as shown in Fig. 3. These findings strongly imply that amplification of *EPSPS* plays a significant role in conferring glyphosate resistance within the MB and TA *A. palmeri* populations. Notably, we observed no instances of an increased *EPSPS* copy number in any of the WT individuals.

### 3.4 *EPSPS* eccDNA sequence analysis

**Figure 4:**
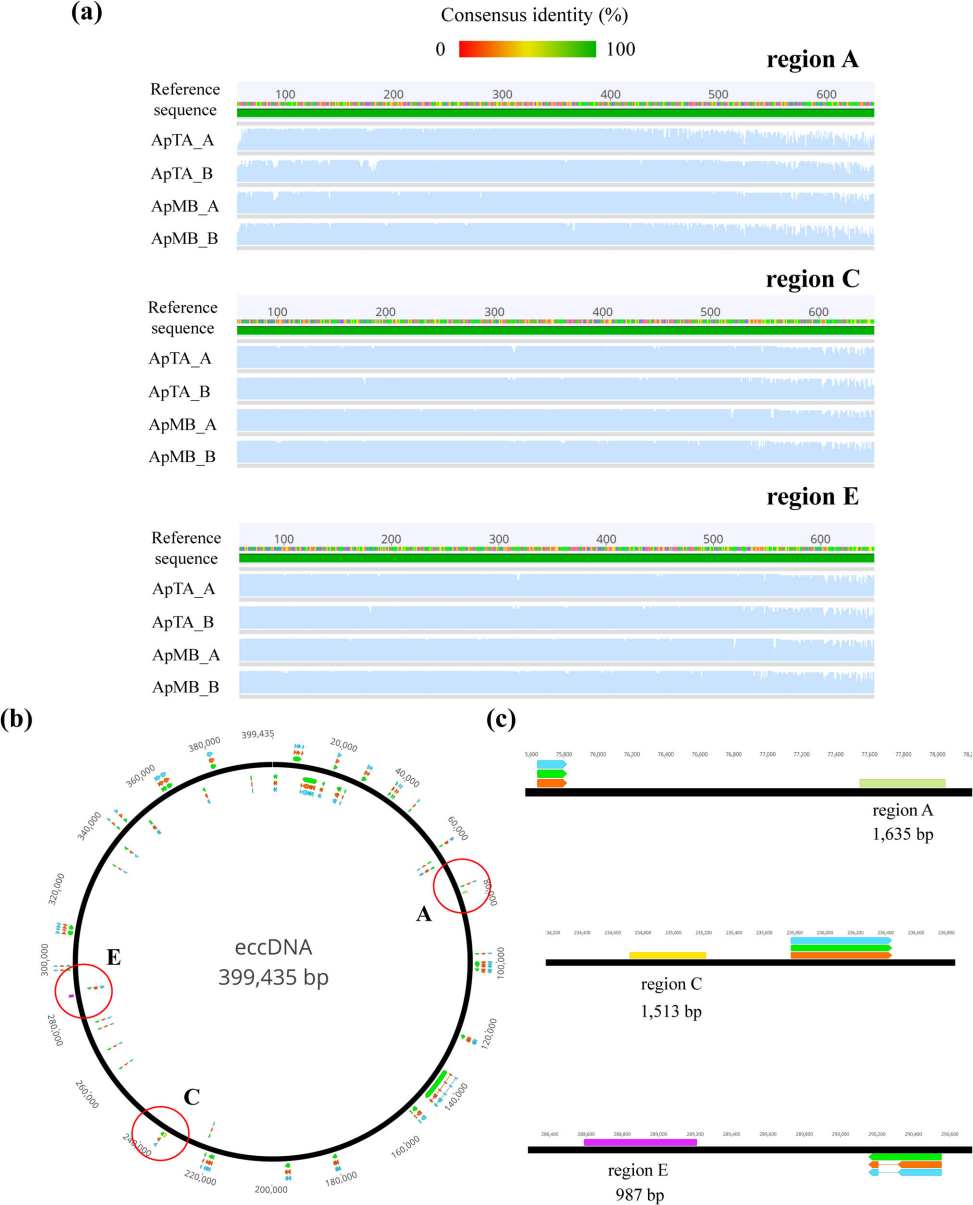
(a) Clustal Omega alignment of the EPSPS cassette reference sequence, with four different glyphosate‐resistant *A. palmeri* Spanish biotypes. Each sequence originates from distinct mother plants (A and B) within populations TA (ApTA) and MB (ApMB). The percentage of identity is represented with a color bar at the top of the sequences. The identity percentage for each nucleotide tends to be closer to 0 (presence of SNPs) as the bar becomes more red, and closer to 100 (analogous to the reference) as it becomes more green. (b) EPSPS cassette from *A. palmeri*, identified as NCBI accession no. MT025716.1. The total sequence length is centrally indicated and the three amplificated region ‘A’, ‘C’, and ‘E’ are marked with a red circle. (c) Close‐up view of the three amplified regions, each located away from a codification sequence, which are annotated in orange, green and blue to represent genes, hypothetical proteins and hypothetical protein mRNA, respectively. Images generated using geneious prime 2023.2.1.

Resistance to glyphosate has been associated with the presence of extrachromosomal circular DNA (eccDNA) called *EPSPS cassette* in *A. palmeri* resistant populations.^36^, ^39^ Among the TA and MB populations we identified that all individual plants that survived a single dose of glyphosate carried this eccDNA. We successfully sequenced a total of 2500 bp from three different regions of the *EPSPS* cassette sequences [A, C and E; Fig. 4(b),(c)]. For region A (1635 bp), a 1335‐bp segment was sequenced with good quality, but the remaining 300 bp could not be analyzed as a consequence of repetitive sequences causing primers to bind to multiple sites during Sanger sequencing. For region C (1513 bp), ≈800 bp were sequenced with the reverse eccC_R primer whereas no sequence was obtained using the eccC_F likely because of the presence of homopolymeric or repetitive sequences leading to weak or absent signals. Lastly, all 987 bp for region E were sequenced successfully. Remarkably, the sequences across populations displayed complete identity, encompassing all three fragments generated by the respective sets of primers. No SNPs were detected in any fragment when compared to the reference genome [Fig. 4(a)]. The consistent lack of sequence variability and the high degree of identity observed among resistant populations in the eccDNA analysis strongly indicates that the *EPSPS* cassette remains highly conserved among these geographically distant populations.

## 4 DISCUSSION

### 4.1 Resistance patterns to ALS‐ and EPSPS‐inhibiting herbicides

Herbicide resistance traits are often associated with an independent evolutionary event driven by herbicide selection; however, the impact of gene flow through pollen or seeds on the establishment of resistance can be equally significant or even greater. Although the dispersion of herbicide‐resistant *A. palmeri* populations in the American continent has been studied, we have limited information about the origin of herbicide resistance for this species in Europe.^44^, ^45^

Plant bioassays combined with molecular studies and DNA sequencing confirmed that two Spanish populations of *A. palmeri* have multiple resistance to ALS and EPSPS inhibitors. The % of plant survival to the ALS‐inhibitor THIF were higher than resistance to IMA in both populations, indicating low frequency of mutations knowing to confer cross‐resistance to sulfonylureas and imidazolinones in each Spanish population [Fig. 1(a)]. A higher survival rate to IMA was reported in TA compared to MB, suggesting different target‐site mutations and/or frequencies between populations (Table 2).

The frequency of mutations known to confer cross‐resistance in this species was low in MB and moderate in TA (Table 2).^46^, ^47^ In fact, one of 20 plants from MB and 10 of 23 plants from TA showed the Trp‐574‐Leu mutation. Another mutation known to confer cross‐resistance to both ALS chemical family is Asp‐376‐Glu,^48^ which was found in just five plants from MB and was absent in survival plants from TA. However, it also is important to stress that recent studies indicate that this mutation confers very low resistance to IMA compared to other imidazolinones as reported previously for *A. retroflexus*.^49^ Surprisingly, the majority (four out of five) also had the Pro‐197‐Thr. This shows an accumulation of multiple mutations in the *ALS* gene at the plant level. Previous studies have noted the coexistence of diverse allele mutations within the same plant.^11^, ^23^, ^29^

In the MB population, most surviving plants had a point mutation at Pro‐197 including two different amino acid substitutions, Pro‐197‐Ser and Pro‐197‐Thr. However, surviving plants from TA population show at the same codon a higher number of amino acid substitutions including: ‐Ser, ‐Thr, ‐Ala and ‐Ile (Table 2). All of the amino acid substitutions at Pro‐197 described in TA and MB populations provide robust resistance to sulfonylureas, and very low or no resistance to imidazolinones.^3^, ^9^, ^11^, ^50^ Mutations at the Pro‐197 position are common in *A. palmeri* as well as in other resistant weed species, such as redroot pigweed (*Amaranthus retroflexus*), common ragweed (*Ambrosia artemisiifolia* L.) and kochia (*Bassia scoparia*).^47^, ^49^, ^51^, ^52^ Finally, the mutation Pro‐197‐Ile identified in population TA, was reported only once previously in *Sisymbrium orientale* and in a population from China, representing a new mutation in the *ALS* gene for *A. palmeri* from Europe.^42^, ^53^, ^54^

Overall, because TA and MB populations were found in areas where ALS‐based herbicides are not applied and therefore, no selection pressure exists, the high number of allelic variants of the *ALS* gene found in TA and MB populations (a total of nine considering plants with double mutations) further indicates recurrent introductions of *A. palmeri* in Europe, and we can speculate that the different mutated *ALS* alleles are generated *ex situ*, in the country of origin.^30^ Although the spread of herbicide resistance in *A. palmeri* could potentially result from pollen‐ or seed‐mediated gene flow from nearby infested areas, this hypothesis can be excluded in this case.^45^

First, there is no presence of resistant *A. palmeri* populations close to those areas where TA and MB populations were collected, making a scenario of resistance gene migration to roadsides through pollen‐mediated gene flow improbable. In fact, ALS inhibitors are not utilized for roadside weed control in Spain to the best of our knowledge, where a combination of glyphosate and mechanical methods is employed instead (personal communication from Plant Health Service of Catalunya).

In order to further understand the resistance mechanisms in the Spanish *A*.

*palmeri* populations, a dose–response study with glyphosate revealed the presence of resistant individuals in both MB and TA populations able to survive to 400 g ae ha^−1^ (few to 800 g ae ha^−1^) of commercial product, whereas the WT was completely controlled (Fig. 1). Our results suggest that increased *EPSPS* copy number is the primary resistance mechanism in both MB and TA populations. Sanger sequencing of the *ApEPSPS* excluded the presence of point mutations in the target gene sequence. *EPSPS* gene duplication is a relatively common mechanism for glyphosate resistance in *A. palmeri*, as well as in other broadleaf and grass species.^43^ Although most plant species typically have between one to two genomic copies of *EPSPS* per haploid genome (or per subgenome in the case of polyploids), glyphosate‐resistant *A. palmeri* has been reported having between 30 and >100 copies.^12^, ^55^ Tested plants from the Spanish populations had 13 to 123 more copies compared to the WT (Fig. 3), consistent with previous studies on this species.^12^, ^31^, ^56^ The presence of multiple gene copies in both MB and TA populations explains their survival rate to glyphosate. This observation aligns with prior research on the glyphosate‐resistant *A. palmeri* populations from Brazil, USA and Japan in which *A. palmeri* plants possessing >30 *EPSPS* copies demonstrated the ability to survive and resist glyphosate at the recommended application rate.^12^, ^23^, ^41^, ^57^

### 4.2 Multiple resistance to ALS‐ and EPSPS‐inhibiting herbicides

We report the presence of multiple resistant mechanisms to ALS and EPSPS inhibitors at the individual plant level. A total of 10 individual plants per population resistant to glyphosate were found to have multiple mutations in the *ALS* gene in addition to the presence of the *EPSPS* eccDNA (Table 3). Three different mutations were found in positions Pro‐197, Asp‐376 and Trp‐574. Surprisingly, for the Pro‐197 four different amino acid substitutions were confirmed: Pro‐197‐Thr, Pro‐197‐Ser, Pro‐197‐Ala and Pro‐197‐Ile. Two plants with a mutation in this position also presented glutamic for aspartic substitution at codon 376 and one with a leucine for a tryptophan at codon 574 occurring together in the same plants.

The sequential application of EPSPS‐ and ALS‐inhibiting herbicides in glasshouse conditions further confirmed the multiple resistance at plant level in MB and TA populations, as these plants accumulate copy number variation and point mutations as TSR mechanisms to each MoA, as shown before. Because we cannot exclude the presence of other resistance mechanisms, further investigations are needed to assess the presence of nontarget site resistance mechanisms to both ALS and EPSPS inhibitors in these populations. Both populations have no history of selection pressure in roadsides with ALS inhibitors and therefore, the large number of plants found with resistance mechanisms to this MoA suggest that they have been introduced from the country of origin. Although glyphosate resistance could be the result of local selective pressure, we exclude that an independent evolutionary event could have been resulted in the *EPSPS* eccDNA in a Spanish population with a very high sequence similarity to the eccDNA found in American populations, as described in other studies.^34^, ^36^, ^56^ For these reasons we suggest that both herbicide resistance mechanisms found in individual plants in Spanish populations likely appeared before being introduced in the country and rapidly spread, either by seeds or pollen. Further investigations are needed to define the evolutionary events leading to multiple resistance at the plant level.

### 4.3 Introduction of the *EPSPS* cassette

Our results showed that *EPSPS* gene amplification is the main glyphosate resistance mechanism in TA and MB populations. This mechanism is attributed to the activity of *EPSPS eccDNA* that previous works showed emerging as unique evolutionary event and rapidly spreading in different countries.^37^, ^38^, ^56^ Supporting this idea, different studies have demonstrated identical *EPSPS eccDNA* sequences in geographically distant populations in North and South America, likely owing to seed movement. Our data support the idea that a similar scenario is currently occurring in Europe.^23^, ^38^, ^56^

Sanger sequencing analysis was conducted on three distinct noncoding regions (A, C and E) of the eccDNA from individual plants (*n* = 43) of the TA and MB populations which survived a single glyphosate dose. The ≈2500 bp analyzed showed 100% identity with the American reference sequence. The absence of any SNPs across all amplified regions strongly supports the idea that the eccDNA present in TA and MB populations is the same as identified in North and South America.^35^, ^37^ This suggests that the resistance mechanism found in Spanish populations originated from a single evolutionary event, likely in North America, and subsequently spread into Spain. The conservation of the eccDNA sequence also has been reported across multiple US populations and for other countries such as Brazil and Uruguay where the North American population seems to be the source of introduction of glyphosate resistance.^37^, ^38^, ^39^, ^56^ However, based on these results, the exact origin of introduction cannot be accurately established.

Nonetheless, when a herbicide‐resistant population is introduced in a new environment, resistance mechanisms could emerge as the result of an independent evolutionary event. Yet, the contribution of pollen‐ and seed‐mediated gene flow is often equal to or higher than independent evolutionary events.^45^ In fact, we did not find any nucleotide difference in the eccDNA sequence between the American and the Spanish populations. Based on the conservation of the eccDNA sequence found in TA and MB populations, this study proposes that glyphosate resistance was introduced in Spain from North America, and the resistance mechanism in TA and MB is the result of seed‐mediated gene flow. The results of this study, although limited to two populations both originating from the same country and with only a portion of eccDNA sequenced, represent an intriguing first step towards understanding the evolutionary nature of multiple resistance in *A. palmeri* populations documented outside the American continent.

All of these results point to the introduction of herbicide resistance in Spain through seed mediated gene flow. *A. palmeri* resistant populations with resistance to up to eight MoAs have been documented worldwide^10^, ^11^, ^13^, ^15^, ^16^, ^17^, ^18^, ^19^, ^22^ and given the continuous influx of new seeds into Spain and Europe, we cannot exclude the possibility that additional herbicide resistance mechanisms also are being introduced. The multiple introductions of *A. palmeri* individuals carrying pre‐existing resistance to multiple herbicides pose a serious threat to European agriculture.^9^, ^29^ The potential for further spread across Europe raises concerns about yield losses in major crops such as maize, sunflower and sugar beet, where effective postemergence control options are already limited. Moreover, the entire Mediterranean region is particularly affected by climate change, experiencing warmer winters and hot, dry summers.^58^ Rising temperatures may render cooler European regions more suitable for *A. palmeri*, expanding the problem beyond the Mediterranean to the rest of Europe. A coordinated European monitoring and containment program for *A. palmeri*, both in agricultural fields and along roadside margins, is essential to ensure the future effectiveness of chemical control strategies.

## 5 CONCLUSIONS

This study is the first comparing the sequences of the eccDNA between the American *A. palmeri* reference genome and multiple herbicide‐resistant populations from Spain. The lack of SNPs among eccDNA from Spanish populations and a reference sequence from a geographically distant plant (USA) supports the hypothesis of a unique evolutionary origin of glyphosate resistance that is spreading across the world, likely from North America. Furthermore, we confirm multiple resistance to EPSPS and ALS inhibitors at both the population and individual levels in Europe. The high number of allelic variants for the *ALS* gene (eight in total) including Pro‐197‐Ile, which represent the first report for a European population of *A. palmeri*, found in areas without a selective pressure, further supports the hypothesis that these independent evolutionary events were selected in America before recurrently arriving to Spain. The agricultural sector in Spain and other European countries, faces a huge threat from this invasive weed species. The arrival of these and new resistance alleles raises high concerns for managing *A. palmeri* in Europe both in crop and noncrop land, because the risk of selecting herbicide‐resistant plants is immense.

## CONFLICT OF INTEREST

The authors declare that they have no known competing financial interests or personal relationships that could have appeared to influence the work reported in this paper.

## Supporting information


**DATA S1:** Supporting Information.

## Data Availability

The data that support the findings of this study are available from the corresponding authors, Jorge Lozano‐Juste and Alfredo Manicardi, upon request.

## References

[1] Rüegg WT , Quadranti M and Zoschke A , Herbicide research and development: challenges and opportunities. Weed Res 47:271–275 (2007).

[2] Kraehmer H , Laber B , Rosinger C and Schulz A , Focus on weed control: herbicides as weed control agents: state of the art: I. Weed control research and Safener technology: the path to modern agriculture. Plant Physiol 166:1119 (2014). 25104723 10.1104/pp.114.241901 PMC4226364

[3] Powles SB and Yu Q , Evolution in action: plants resistant to herbicides. Annu Rev Plant Biol 61:317–347 (2010). 20192743 10.1146/annurev-arplant-042809-112119

[4] Torra J , Osuna MD , Merotto A and Vila‐Aiub M , Editorial: multiple herbicide‐resistant weeds and non‐target site resistance mechanisms: a global challenge for food production. Front Plant Sci 12:2372 (2021). 10.3389/fpls.2021.763212 PMC8581628 34777445

[5] Kettunen MGP , Gollasch S , Pagad S , Starfinger U , ten Brink P and Shine C , Technical support to EU strategy on invasive alien species (IAS) assessment of the impacts of IAS in Europe and the EU. Institute for European Environmental Policy (IEEP):1–124 (2009).

[6] Paini DR , Sheppard AW , Cook DC , de Barro PJ , Worner SP and Thomas MB , Global threat to agriculture from invasive species. Proc Natl Acad Sci USA 113:7575–7579 (2016). 27325781 10.1073/pnas.1602205113 PMC4941431

[7] Boscutti F , Sigura M , de Simone S and Marini L , Exotic plant invasion in agricultural landscapes: a matter of dispersal mode and disturbance intensity. Appl Veg Sci 21:250–257 (2018).

[8] Mahoney DJ , Jordan DL , Hare AT , Leon RG , Roma‐Burgos N , Vann MC *et al*., Palmer Amaranth (Amaranthus palmeri) growth and seed production when in competition with Peanut and other crops in North Carolina. Agronomy 11:1734 (2021).

[9] Matzrafi M , Scarabel L , Milani A , Iamonico D , Torra J , Recasens J *et al*., Amaranthus palmeri S. Watson: a new threat to agriculture in Europe and the Mediterranean region. Weed Res 6:1–16 (2023).

[10] Gaines TA , Patterson EL and Neve P , Molecular mechanisms of adaptive evolution revealed by global selection for glyphosate resistance. New Phytol 223:1770–1775 (2019). 31002387 10.1111/nph.15858

[11] Singh S , Singh V , Salas‐Perez RA , Bagavathiannan MV , Lawton‐Rauh A and Roma‐Burgos N , Target‐site mutation accumulation among ALS inhibitor‐resistant palmer amaranth. Pest Manag Sci 75:1131–1139 (2019). 30298618 10.1002/ps.5232

[12] Chahal PS , Varanasi VK , Jugulam M and Jhala AJ , Glyphosate‐resistant palmer Amaranth (Amaranthus palmeri) in Nebraska: confirmation, EPSPS gene amplification, and response to POST corn and soybean herbicides. Weed Technol 31:80–93 (2017).

[13] Salas RA , Burgos NR , Tranel PJ , Singh S , Glasgow L , Scott RC *et al*., Resistance to PPO‐inhibiting herbicide in palmer amaranth from Arkansas. Pest Manag Sci 72:864–869 (2016). 26817647 10.1002/ps.4241 PMC5069602

[14] Giacomini DA , Umphres AM , Nie H , Mueller TC , Steckel LE , Young BG *et al*., Two new PPX2 mutations associated with resistance to PPO‐inhibiting herbicides in Amaranthus palmeri. Pest Manag Sci 73:1559–1563 (2017). 28370968 10.1002/ps.4581

[15] Hwang J‐I , Norsworthy JK , Carvalho‐Moore P , Barber LT , Butts TR and JS ME , Exploratory analysis on herbicide metabolism and very‐long‐chain fatty acid production in metolachlor‐resistant palmer Amaranth (Amaranthus palmeri S. Wats.). J Agric Food Chem 71:6014–6022 (2023). 10.1021/acs.jafc.3c00196 37036857

[16] Gossett BJ , Murdock EC and Toler JE , Resistance of palmer Amaranth (Amaranthus palmeri) to the dinitroaniline herbicides. Weed Technol 6:587–591 (1992).

[17] Foster DC and Steckel LE , Confirmation of dicamba‐resistant palmer amaranth in Tennessee. Weed Technol 36:777–780 (2022).

[18] Küpper A , Peter F , Zöllner P , Lorentz L , Tranel PJ , Beffa R *et al*., Tembotrione detoxification in 4‐hydroxyphenylpyruvate dioxygenase (HPPD) inhibitor‐resistant palmer amaranth (Amaranthus palmeri S. Wats.). Pest Manag Sci 74:2325–2334 (2018). 29105299 10.1002/ps.4786

[19] Noguera MM , Porri A , Werle IS , Heiser J , Brändle F , Lerchl J *et al*., Involvement of glutamine synthetase 2 (GS2) amplification and overexpression in Amaranthus palmeri resistance to glufosinate. Planta 256:57 (2022). 35960361 10.1007/s00425-022-03968-2 PMC9374794

[20] Kumar V , Liu R , Boyer G and Stahlman PW , Confirmation of 2,4‐D Resistance and Identification of Multiple Resistance in a Kansas Palmer Amaranth (Amaranthus Palmeri) Population, Pest Management Science **75**:2925–2933. John Wiley & Sons, Ltd (2019). 10.1002/ps.5400 30843341

[21] Chaudhari S , Varanasi VK , Nakka S , Bhowmik PC , Thompson CR , Peterson DE *et al*., Evolution of target and non‐target based multiple herbicide resistance in a single palmer amaranth (Amaranthus palmeri) population from Kansas. Weed Technol 34:447–453 (2020).

[22] Shyam C , Borgato EA , Peterson DE , Dille JA and Jugulam M , Predominance of metabolic resistance in a six‐way‐resistant palmer Amaranth (Amaranthus palmeri) population. Front Plant Sci 11:2162 (2021). 10.3389/fpls.2020.614618 PMC7841332 33519873

[23] Torra J , Royo‐Esnal A , Romano Y , Osuna MD , León RG and Recasens J , Amaranthus palmeri a new invasive weed in Spain with herbicide resistant biotypes. Agronomy 10:1–13 (2020).

[24] Shimono A , Kanbe H , Nakamura S , Ueno S , Yamashita J and Asai M , Initial invasion of glyphosate‐resistant Amaranthus palmeri around grain‐import ports in Japan. Plants People Planet 2:640–648 (2020).

[25] Milani A , Panozzo S , Farinati S , Iamonico D , Sattin M , Loddo D *et al*., Recent discovery of amaranthus palmeri s. Watson in Italy: characterization of als‐resistant populations and sensitivity to alternative herbicides. Sustainability 13:7003 (2021).

[26] Kanatas P , Tataridas A , Dellaportas V and Travlos I , First report of Amaranthus palmeri S. Wats. In cotton, maize and sorghum in Greece and problems with its management. Agronomy 11:1721 (2021).

[27] Mennan H , Kaya‐Altop E , Belvaux X , Brants I , Zandstra BH , Jabran K *et al*., Investigating glyphosate resistance in Amaranthus palmeri biotypes from Turkey. Phytoparasitica 49:1043–1052 (2021).

[28] Gaines TA , Slavov GT , Hughes D , Küpper A , Sparks CD , Oliva J *et al*., Investigating the origins and evolution of a glyphosate‐resistant weed invasion in South America. Mol Ecol 30:5360–5372 (2021). 34637174 10.1111/mec.16221

[29] Manicardi A , Scarabel L , Llenes JM , Montull JM , Osuna MD , Farré JT *et al*., Genetic basis and origin of resistance to acetolactate synthase inhibitors in Amaranthus palmeri from Spain and Italy. Pest Manag Sci 79:4886–4896 (2023). 37515753 10.1002/ps.7690

[30] Beckie HJ , Busi R , Bagavathiannan MV and Martin SL , Herbicide resistance gene flow in weeds: under‐estimated and under‐appreciated. Agric Ecosyst Environ 283:106566 (2019).

[31] Kniss AR , Genetically engineered herbicide‐resistant crops and herbicide‐resistant weed evolution in the United States. Weed Sci 66:260–273 (2018).

[32] Heap I , Global perspective of herbicide‐resistant weeds. Pest Manag Sci 70:1306–1315 (2014). 24302673 10.1002/ps.3696

[33] Baucom RS , Evolutionary and ecological insights from herbicide‐resistant weeds: what have we learned about plant adaptation, and what is left to uncover? New Phytol 223:68–82 (2019). 30710343 10.1111/nph.15723

[34] Wise AM , Grey TL , Prostko EP , Vencill WK and Webster TM , Establishing the geographical distribution and level of acetolactate synthase resistance of palmer Amaranth (Amaranthus palmeri) accessions in Georgia. Weed Technol 23:214–220 (2009).

[35] Nakka S , Thompson CR , Peterson DE and Jugulam M , Target site–based and non–target site based resistance to ALS inhibitors in palmer Amaranth (Amaranthus palmeri). Weed Sci 65:681–689 (2017).

[36] Larran AS , Palmieri VE , Perotti VE , Lieber L , Tuesca D and Permingeat HR , Target‐site resistance to acetolactate synthase (ALS)‐inhibiting herbicides in Amaranthus palmeri from Argentina. Pest Manag Sci 73:2578–2584 (2017). 28703943 10.1002/ps.4662

[37] Küpper A , Borgato EA , Patterson EL , Netto AG , Nicolai M , Carvalho SJP *et al*., Multiple resistance to glyphosate and acetolactate synthase inhibitors in palmer amaranth (amaranthus palmeri) identified in Brazil. Weed Sci 65:317–326 (2017).

[38] Reinhardt C , Vorster J , Küpper A , Peter F , Simelane A , Friis S *et al*., A nonnative palmer amaranth (Amaranthus palmeri) population in the Republic of South Africa is resistant to herbicides with different sites of action. Weed Sci 70:183–197 (2022).

[39] Baruch R and Matzrafi M , Weed Management in Israel Challenges and Approaches. Weed Science in the Asian‐Pacific Region, India, pp. 253–270 (2015).

[40] Gaines TA , Zhang W , Wang D , Bukun B , Chisholm ST , Shaner DL *et al*., Gene amplification confers glyphosate resistance in Amaranthus palmeri. Proc Natl Acad Sci USA 107:1029–1034 (2010). 20018685 10.1073/pnas.0906649107 PMC2824275

[41] Vila‐Aiub MM , Fitness of herbicide‐resistant weeds: current knowledge and implications for management. Plan Theory 8:1–11 (2019). 10.3390/plants8110469 PMC6918315 31683943

[42] Chtourou M , Osuna MD , Vázquez‐García JG , Lozano‐Juste J , de Prado R , Torra J *et al*., Pro197Ser and the new Trp574Leu mutations together with enhanced metabolism contribute to cross‐resistance to ALS inhibiting herbicides in Sinapis alba. Pestic Biochem Physiol 201:105882 (2024). 38685248 10.1016/j.pestbp.2024.105882

[43] Yu Q and Powles SB , Resistance to AHAS inhibitor herbicides: current understanding. Pest Manag Sci 70:1340–1350 (2014). 24338926 10.1002/ps.3710

[44] Molin WT , Wright AA , Lawton‐Rauh A and Saski CA , The unique genomic landscape surrounding the EPSPS gene in glyphosate resistant Amaranthus palmeri: a repetitive path to resistance. BMC Genomics 18:91 (2017). 28095770 10.1186/s12864-016-3336-4 PMC5240378

[45] Koo DH , Molin WT , Saski CA , Jiang J , Putta K , Jugulam M *et al*., Extrachromosomal circular DNA‐based amplification and transmission of herbicide resistance in crop weed Amaranthus palmeri. Proc Natl Acad Sci USA 115:3332–3337 (2018). 29531028 10.1073/pnas.1719354115 PMC5879691

[46] Molin WT , Yaguchi A , Blenner M and Saski CA , The EccDNA replicon: a heritable, extranuclear vehicle that enables gene amplification and glyphosate resistance in Amaranthus palmeri. Plant Cell 32:2132–2140 (2020). 32327538 10.1105/tpc.20.00099 PMC7346551

[47] Manicardi A , Milani A , Scarabel L , Mora G , Recasens J , Llenes JM *et al*., First report of glyphosate resistance in an Amaranthus palmeri population from Europe. Weed Res 65:1–6 (2023).

[48] Tranel PJ , Wright TR and Heap IM , Mutations in herbicide‐resistant weeds to ALS inhibitors (2025). http://wwwweedsciencecom Accessed 5 May 2025.

[49] Molin WT , Wright AA , VanGessel MJ , McCloskey WB , Jugulam M and Hoagland RE , Survey of the genomic landscape surrounding the 5‐enolpyruvylshikimate‐3‐phosphate synthase (EPSPS) gene in glyphosate‐resistant Amaranthus palmeri from geographically distant populations in the USA. Pest Manag Sci 74:1109–1117 (2018). 28686355 10.1002/ps.4659

[50] Molin T , Patterson EL and Saski CA , Homogeneity among glyphosate‐resistant Amaranthus palmeri in geographically distant locations. PLoS One 15:e0233813 (2020). 32903277 10.1371/journal.pone.0233813 PMC7480871

[51] Camposano HS , Molin WT and Saski CA , Sequence characterization of eccDNA content in glyphosate sensitive and resistant palmer amaranth from geographically distant populations. PLoS One 17:e0260906 (2022). 36103503 10.1371/journal.pone.0260906 PMC9473621

[52] Chandi A , Milla‐Lewis SR , Giacomini D , Westra P , Preston C , Jordan DL *et al*., Inheritance of evolved glyphosate resistance in a North Carolina palmer Amaranth (Amaranthus palmeri) biotype. Int J Agron 2012:1–7 (2012).

[53] García MJ , Palma‐Bautista C , Rojano‐Delgado AM , Bracamonte E , Portugal J , Alcántara‐de la Cruz R *et al*., The triple amino acid substitution TAP‐IVS in the EPSPS gene confers high glyphosate resistance to the superweed Amaranthus hybridus. Int J Mol Sci 20:2396 (2019). 31096560 10.3390/ijms20102396 PMC6567628

[54] Ji M , Yu H , Cui H , Chen J , Yu J and Li X , A new Pro‐197‐Ile mutation in *Amaranthus palmeri* associated with acetolactate synthase‐inhibiting herbicide resistance. Plan Theory 14:525 (2025). 10.3390/plants14040525 PMC11859721 40006784

[55] Darmency H , Movement of resistance genes among plants. ACS Symp Ser 645:209–220 (1996).

[56] Dweikat IM , Gelli M , Bernards M , Martin A and Jhala A , Mutations in the acetolactate synthase (ALS) enzyme affect shattercane (Sorghum bicolor) response to ALS‐inhibiting herbicides. Hereditas 160:28 (2023). 37344897 10.1186/s41065-023-00291-y PMC10283220

[57] Gaines TA , Duke SO , Morran S , Rigon CAG , Tranel PJ , Küpper A *et al*., Mechanisms of evolved herbicide resistance. J Biol Chem 295:10307–10330 (2020). 32430396 10.1074/jbc.REV120.013572 PMC7383398

[58] Elham A , Cramer W , Carnicer J , Georgopoulou E , Himli N , Le Cozannet G *et al*., Climate Change 2022: Impacts, Adaptation and Vulnerability. Cambridge University Press, UK, pp. 2233–2272 (2022).

