# Supplementary material for: Analysis of multiple‐herbicide resistant Amaranthus palmeri populations from Spain points to an introduction of the eccDNA from America
Source: Pest Manag Sci. 2025 Jul 10;81(10):6807–19. doi: 10.1002/ps.70034 (PMC12441762; doi:10.1002/ps.70034)
Supplement: Supplementary file 1 — DATA S1: Supporting Information. [file PS-81-6807-s001.docx]

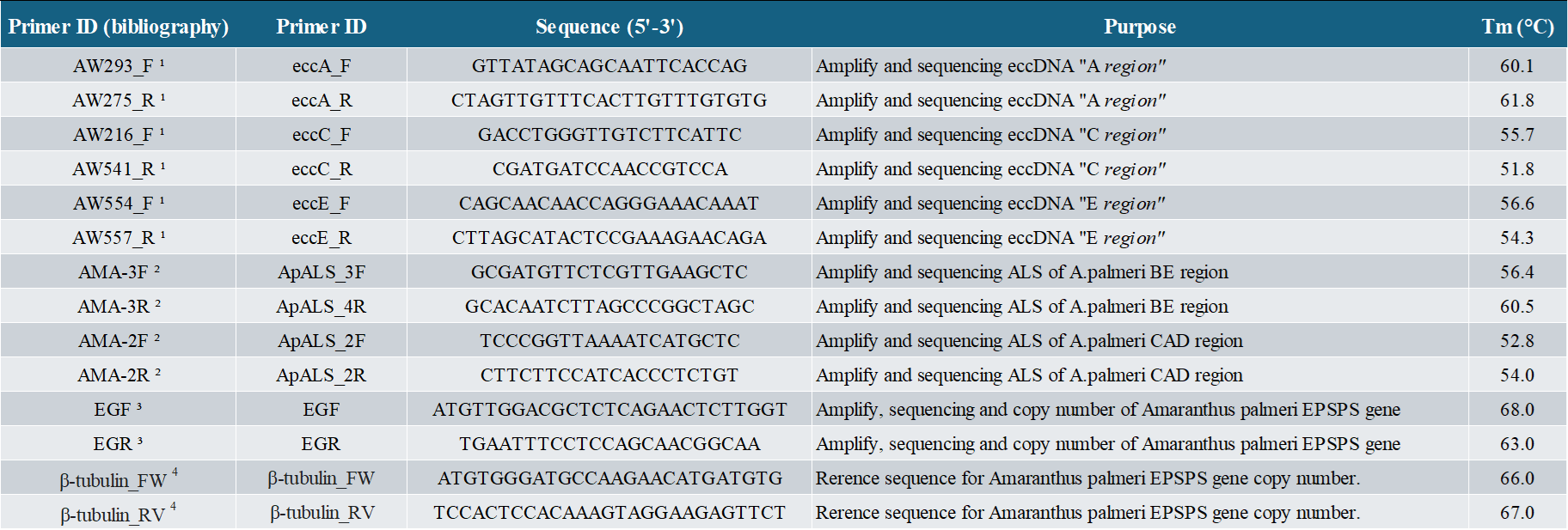


Table S1: all the primers used for the partial ALS, EPSPS and eccDNA amplification and sanger sequencing and for the relative EPSPS gene copy number variation. The primer ID present in the bibliography is reported in the first column, while the IDs used in the present work are in the second column. TM (°C) means the primers melting temperature. ^1^(Molin et al., 2018) ^2^(Scarabel et al., 2007) ^3^(Gaines et al., 2010) ^4^(Godar et al., 2015)

| **Process** | **Amplified gene sequence** | | |
| --- | --- | --- | --- |
|  | ALS *CAD* | ALS *BE* | EPSPS |
| Denaturation | 95°C for 30s | | |
| Anelling | 58°C for 30 s | 58°C for 30 s | 61°C for 30 s |
| Extension | 72°C for 54 s | 72°C for 21 s | 72°C for 15 s |
| Number of cycles | 35 | | |

Table S2: the conditions for each PCR reaction corresponding to the amplified gene sequences. “CAD” and “BE” referred to the two different ALS regions in which the most common reported mutations were found.

| **Process** | **Steps** | **Temperature** | **Time** | **Nr. Cycles** |
| --- | --- | --- | --- | --- |
| PCR | Denaturation | 95°C | 15 s | 40 |
|  | Anelling | 61°C | 1 m |  |
| Melt Curve | Step 1 | 95°C | 15 s | N/A |
|  | Step 2 | 61°C | 1 m |  |
|  | Step 3 | 0.1°C/s up to 95°C | |  |

Table S3: The conditions used in qPCR reactions to compute the relative EPSPS copy number variation.
